# Supplementary material for: Neonatally imprinted stromal cell subsets induce tolerogenic dendritic cells in mesenteric lymph nodes
Source: Nat Commun. 2018 Sep 25;9:3903. doi: 10.1038/s41467-018-06423-7 (PMC6156403; doi:10.1038/s41467-018-06423-7)
Supplement: Supplementary file 1 — Supplementary Information [file 41467_2018_6423_MOESM1_ESM.pdf]

Supplementary Information

Neonatally imprinted stromal cell subsets induce tolerogenic dendritic cells in mesenteric lymph nodes

Pezoldt, Pasztoi, et al.

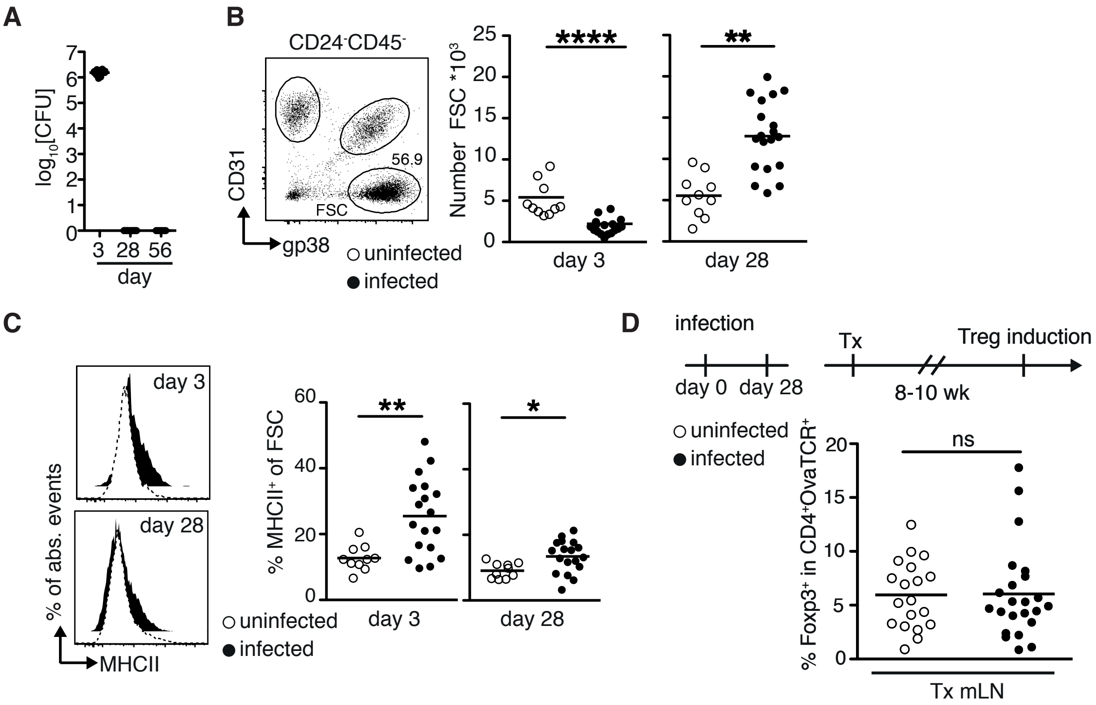

Supplementary Figure 1

Supplementary Figure 1: Impact of *Yersinia pseudotuberculosis* infection and chronic DSS colitis on tolerogenic properties of gut-draining LNs. Seven to eight week old BALB/c mice were orally infected with 10^9 CFU *Yersinia pseudotuberculosis*. (**A**) Scatterplot of bacterial load in mLNs of infected mice at indicated time points p.i.. (**B**) Representative dotplot shows viable $CD24^-CD45^-$ cells from mLNs of an uninfected control mouse. Number indicates frequency of FSCs. Scatterplots summarize numbers of mLN FSCs defined as viable $CD24^-CD45^-CD31^-gp38^+$ cells at indicated time points p.i. (open circles, uninfected; filled circles, infected). Data were pooled from two independent experiments (n = 10-19). (**C**) Representative histogram overlay depicts MHCII expression on gated FSCs (open histogram, uninfected; filled histogram, infected). Scatterplots summarize frequencies of $MHCII^+$ mLN FSCs at indicated time points p.i. (open circles, uninfected; filled circles, infected). Data were pooled from two independent experiments (n = 10-19). (**D**) mLNs of mice day 28 p.i. and of uninfected control mice were transplanted. Eight to ten weeks later, transplanted mice received CPDviolet-labeled cells isolated from $Foxp3^{hCD2}xRag2^{-/-}xDO11.10$ mice. On two consecutive days, recipients were immunized via repetitive *i.v.* injection of $Ova_{323-339}$ peptide and analyzed on day 3 after the first immunization. Scatterplot summarizes frequencies of *de novo* induced $Foxp3^+$ Tregs among transferred $OvaTCR^+CD4^+$ cells recovered from transplanted mLNs (open circles, uninfected; filled circles, infected). Data pooled from two independent experiments are shown (n = 10-23). p.i., post infection; CFU, colony-forming unit; FSC, fibroblastic stromal cells; Tx, transplantation.

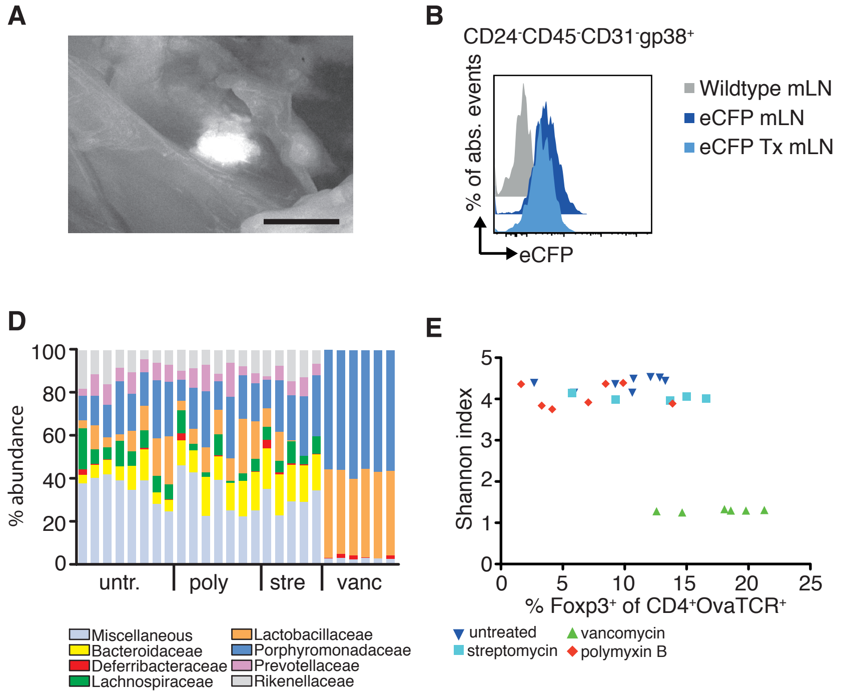

Supplementary Figure 2

Supplementary Figure 2: Engraftment of neonatal mLN stromal cells and microbiota composition in antibiotic-treated mice. (**A-B**) Neonatal mLNs from Actβ-eGFP or Actβ-eCFP reporter mice were transplanted into the popliteal fossa of C57BL/6 mice. Nine to ten weeks later, transplanted mice were analyzed (**A**) Exemplary fluorescence microscopy picture of engrafted neonatal mLN. Scale bar = 2 mm. (**B**) Transplanted mLNs were excised and three mLNs were pooled to isolate FSCs for flow cytometric analysis. Endogenous mLNs from wildtype and Actβ-eCFP mice served as controls. Histograms depict eCFP-fluorescence intensity on gated $CD24^{-}CD45^{-}CD31^{-}gp38^{+}$ FSCs from transplanted mLNs, endogenous mLNs of wildtype mice and endogenous mLNs of Actβ-eCFP mice. Representative results from three independently performed experiments are depicted. (**C-E**) Mothers and their offspring were treated with antibiotics starting seven days post conception until four weeks of age. Untreated mice served as controls. mLNs of continuously antibiotics-treated offspring were transplanted to the popliteal fossa of SPF-housed mice. In parallel, fecal samples of LN donor mice were sampled for analysis of 16S rDNA of the V1-2 region. After at least 10 weeks of reconstitution, transplanted mice received CPDviolet-labeled cells isolated from $Foxp3^{hCD2}xRag2^{-/-}xDO11.10$ mice, followed by *i.v.* injection of $Ova_{323-339}$ peptide on two consecutive days. Cells from transplanted LNs were analyzed on day 3 after the first immunization. Data pooled from two independent experiments are shown (n = 5-7). (**C**) NMDS plot of 16S rDNA analysis of the V1-2 region. (**D**) Absolute abundance of family based on 16S rDNA analysis. (**E**) Treg induction in transplanted mLNs and corresponding Shannon index of bacterial diversity. poly, polymyxin B; strep, streptomycin; Tx, transplantation; untr., untreated; vanc, vancomycin.

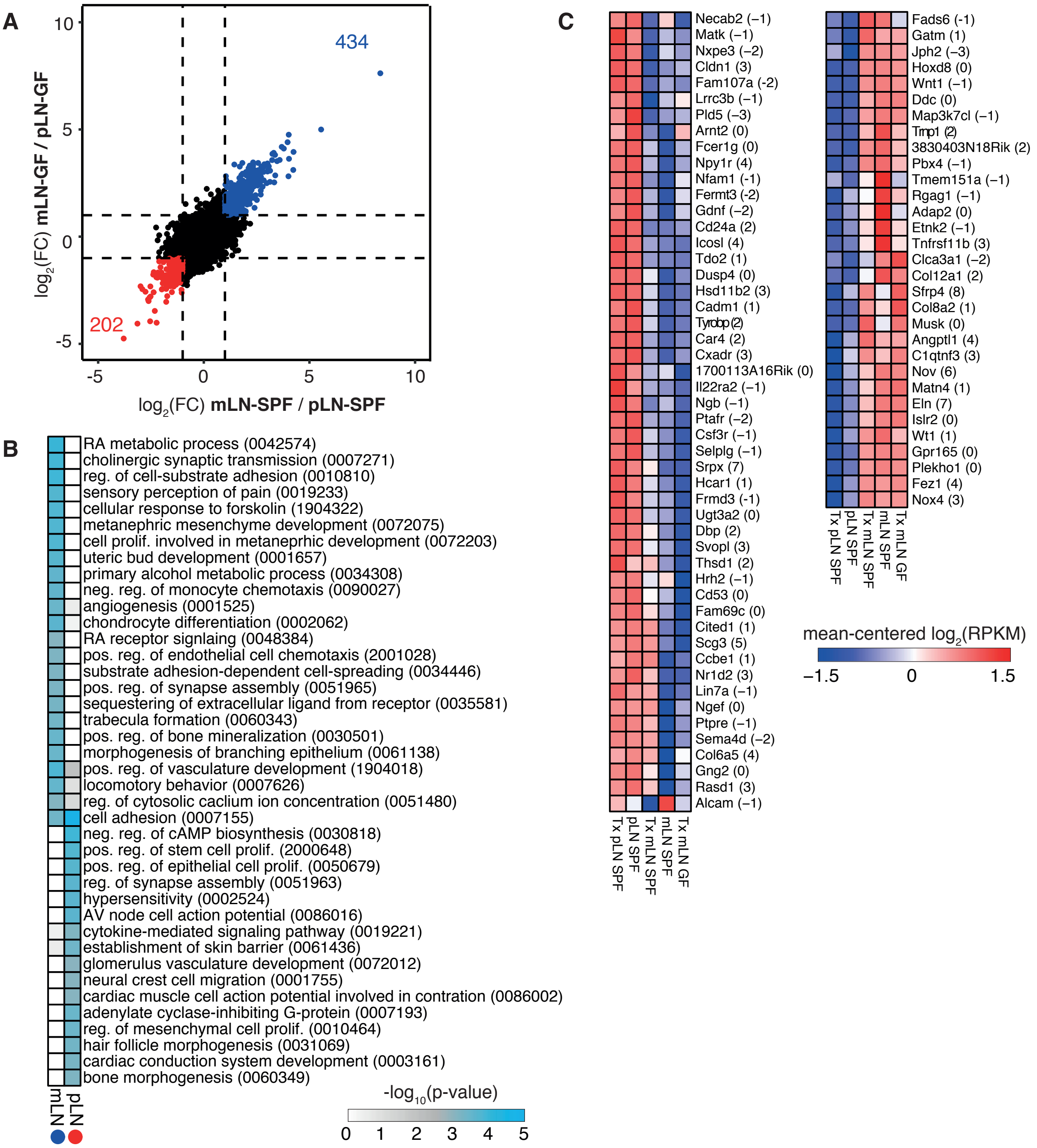

Supplementary Figure 3

Supplementary Figure 3: Impact of microbiota on the tissue-specific transcriptional signatures of FSCs. (**A-B**) $CD45^-Ter119^-CD31^-gp38^+$ FSCs were isolated from mLNs and pLNs of GF or SPF mice, and RNA-seq was performed. (**A**) Colored numbers in scatterplots represent DEGs for the respective pair-wise comparisons of FSCs with $|log_2(FC)| \geq 1$ and q-value ≤ 0.05 for both indicated comparisons. FC-plot shows location-dependent differential expression in the presence (x-axis) versus absence (y-axis) of commensals. (**B**) GO analysis of biological processes of DEGs up-regulated in mLN (blue dot) or pLN (red dot) independent of commensal colonization. Data pooled from two independent experiments. **(C**) $CD45^-CD24^-CD31^-gp38^+$ FSCs were isolated from endogenous mLN- and pLN-SPF, transplanted pLN- and mLN-SPF or transplanted mLN-GF. RNA-seq[L] and subsequent analysis were performed. A heatmap showing expression of genes persistently up- or down-regulated in both transplanted mLN-GF and mLN-SPF as compared to endogenous pLN-SPF was generated. Numbers in brackets indicate average $log_2(RPKM)$ expression of respective gene across all experimental groups. FC, fold-change; GO, Gene Ontology; RPKM, reads per kilobase of exon length per million mapped reads.

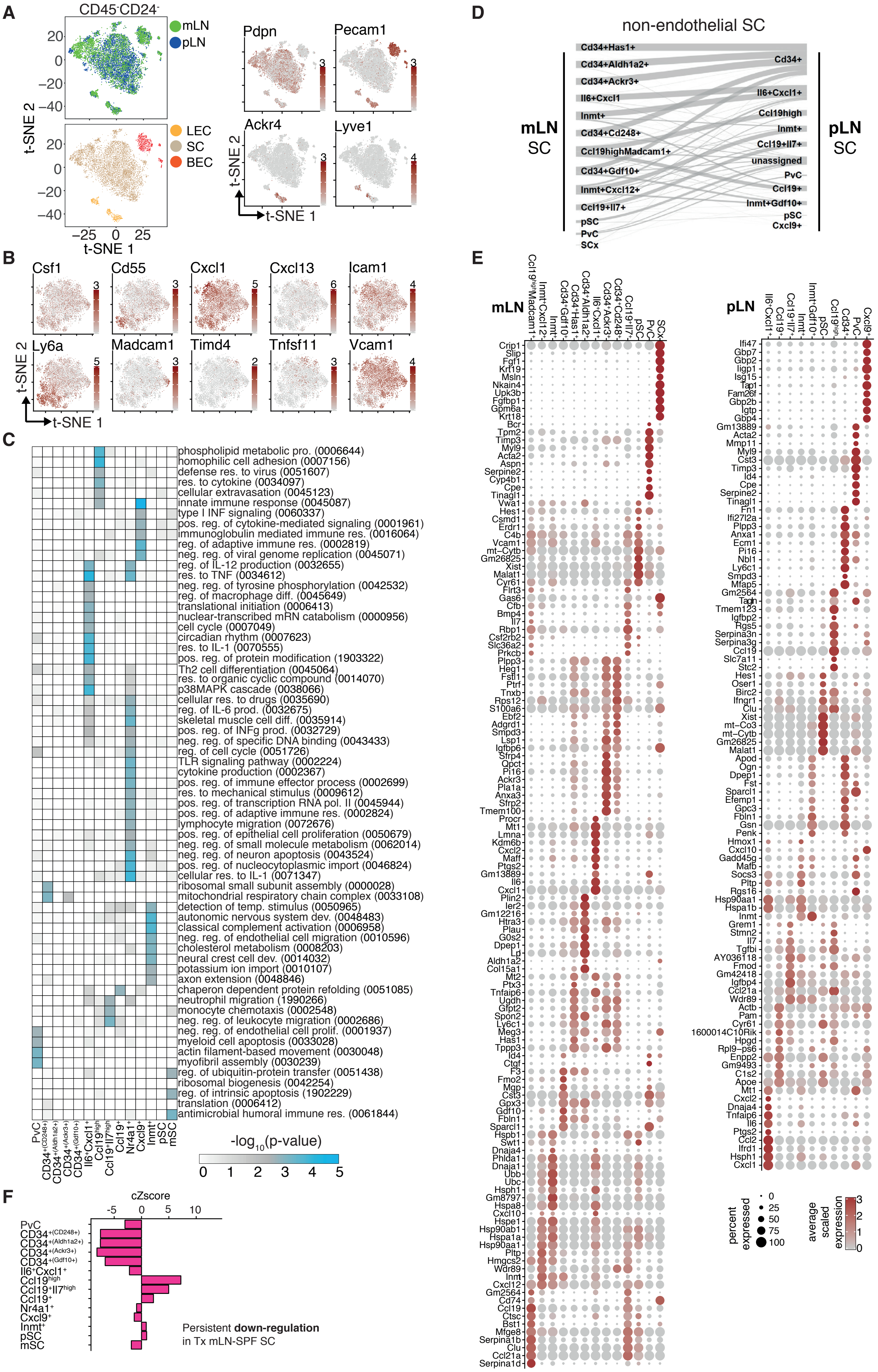

Supplementary Figure 4

Supplementary Figure 4: scRNA-seq reveals common SC subsets across non-endothelial SCs from mLN and pLN. Single cell suspensions from mLNs and pLNs were sorted for CD24^{-}CD45^{-} cells and subjected to scRNA-seq, yielding quality-controlled transcriptomes of 9257 cells. Non-endothelial SCs were identified as non-LECs, non-BECs and *Pecam1*$^{-}$ and *Ackr4*$^{-}$. (**A**) t-SNE plot of merged pLN and mLN stromal cells showing cluster segregation, overlay of mLN and pLN cells and expression of key LN stromal cell associated genes across all CD24^{-}CD45^{-} cells on t-SNE plot. (**B**) Expression of selected DEGs across all non-endothelial SCs on t-SNE plot. (**C**) GO analysis of biological processes for up-regulated DEGs across the non-endothelial SC clusters as identified in Fig. 4A; numbers refer to GO identifiers. (**D**) Projection of subset specific transcriptional signatures from mLN onto pLN on a cell-to-cell basis using *scmap*. (**E**) Dotplot of top 10 DEGs per cluster with highest fold-change in expression for pLN and mLN across all clusters per organ. (**F**) Bargraph shows cumulative Z-score (cZscore) per cluster for the genes persistently down-regulated in FSCs from transplanted mLN-SPF. Data pooled from two independent experiments. BEC, blood endothelial cell; DEG, differentially expressed gene; GO, gene ontology; LEC, lymphatic endothelial cell; mSC, metabolic stromal cells; neg., negative; pos., positive; pro., process; prod., production; pSC, proliferating stromal cells; PvC, perivascular cells; reg., regulation; res., response; SC, stromal cell; temp., temperature; t-SNE, t-distributed stochastic neighbour embedding.

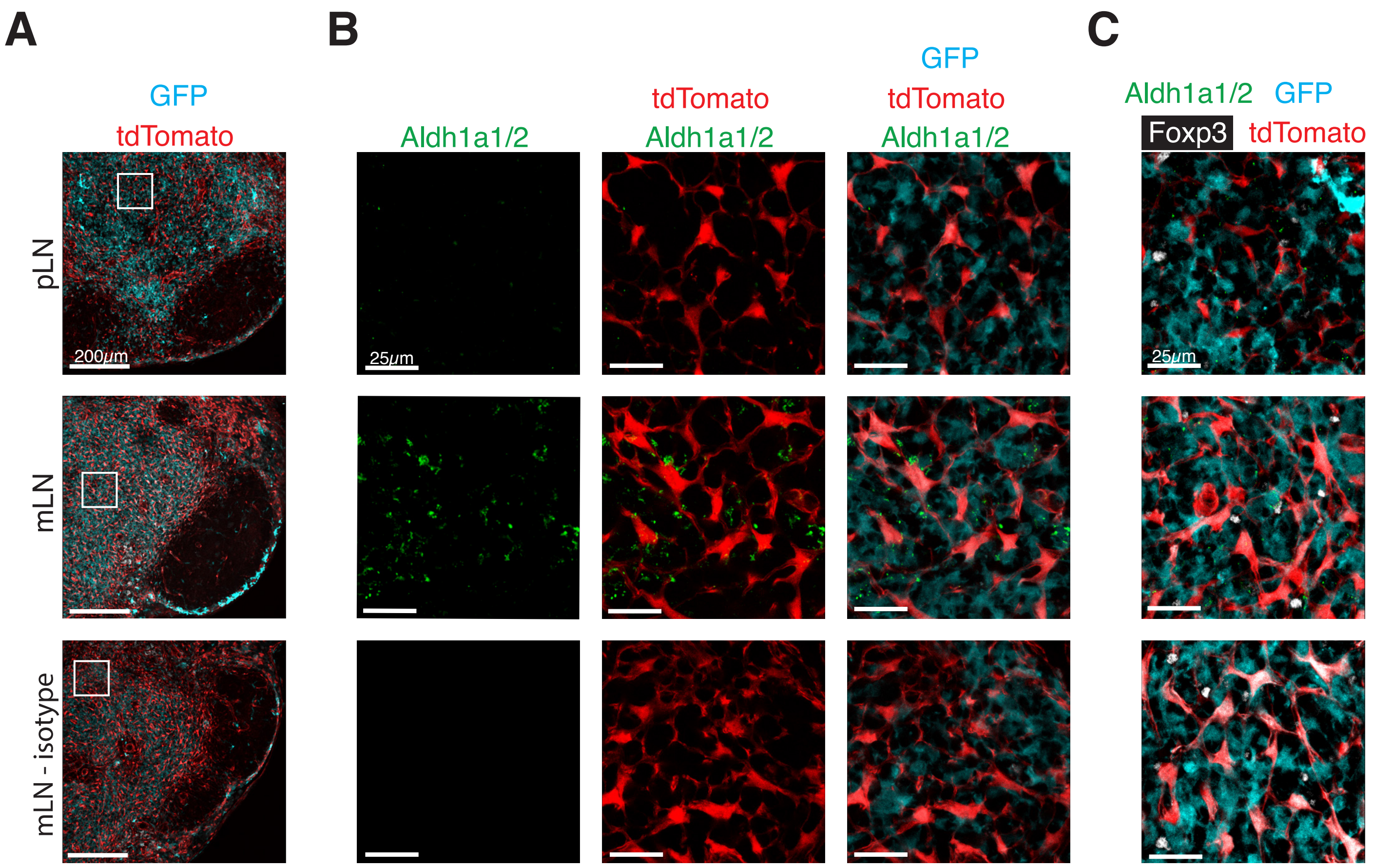

Supplementary Figure 5

Supplementary Figure 5: Expression of Aldh1a2 is limited to DCs in the T cell zone of the mLN. *Ccl19*^{Cre}x*Rosa*^{tdT} mice were fully irradiated and reconstituted with total BM cells isolated from *Zbtb46*$^{gfp/+}$ mice. Forty days later, mLNs and pLNs from chimeric mice were sectioned and stained for ALDH1A1-antibody (cross-reactive to ALDH1A2) or a corresponding isotype control, and Foxp3. Images were obtained using confocal microscopy. (**A**) Low magnification views of pLN and mLN parenchymas. Bars: 200 µm. (**B**) High-magnification views of tdT^{+} LN stromal cells, GFP^{+} DCs and Aldh1a1/2 expression in the T cell zone from the corresponding inserts indicated in (A). Bars: 25 µm. (**C**) Confocal imaging of Foxp3^{+} cells in pLN and mLN T cell area. Bars: 25 µm. Data are representative of one experiment. tdT, tdTomato.

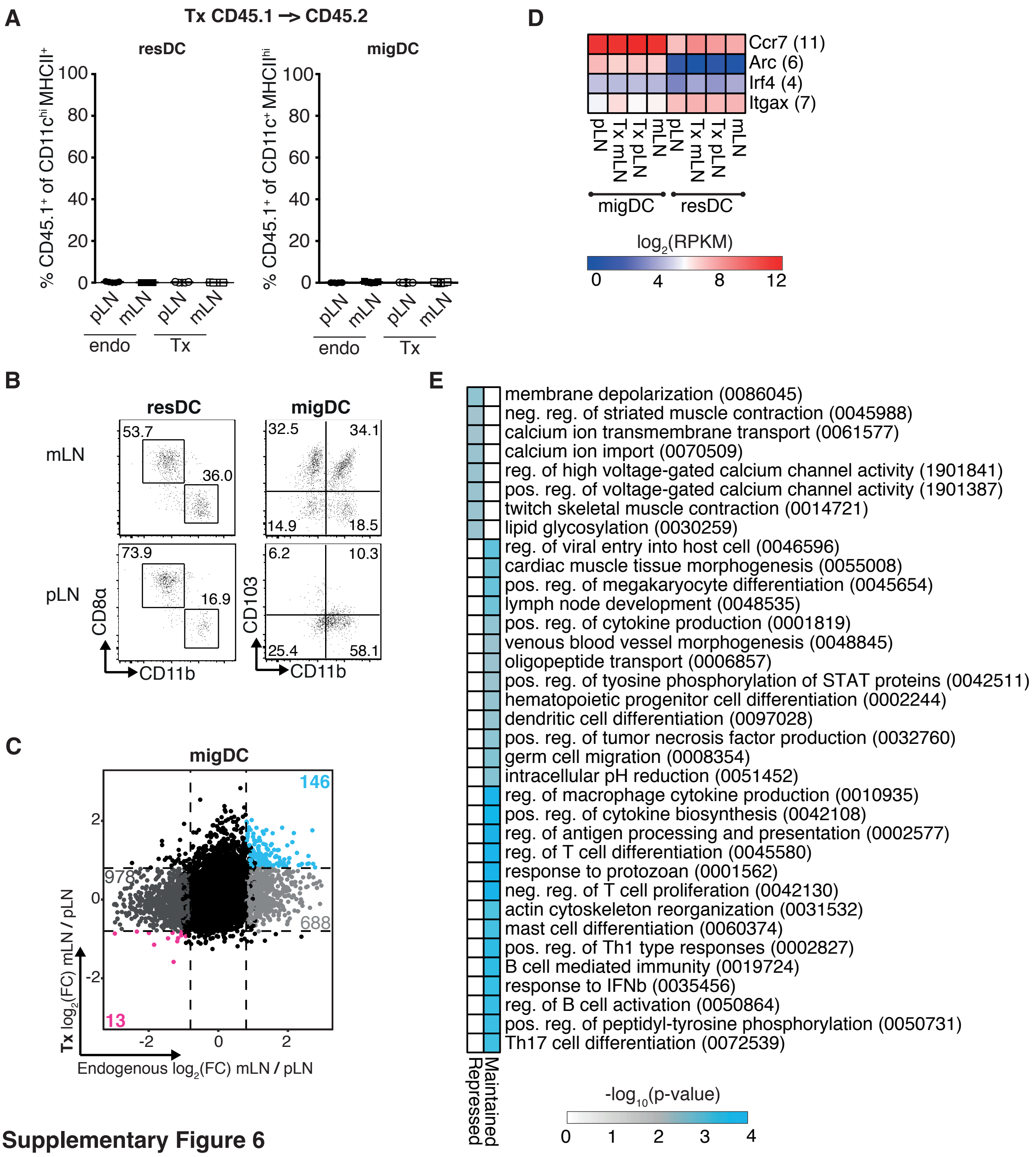

Supplementary Figure 6

Supplementary Figure 6: Frequency of donor-derived DCs in transplanted LNs, DC subset composition in endogenous LNs and transcriptional signature of migDCs from endogenous and transplanted LNs. (**A**) CD45.1 mice served as donors for LNs transplanted to the popliteal fossa of CD45.2 recipient mice. Eight to sixteen weeks later, transplanted (Tx) and endogenous (endo) LNs were resected, single cell suspensions generated by enzymatic digestion, and the frequency of $CD45.1^+$ cells among resDCs and migDCs tested by flow cytometry. Scatterplots summarize frequency of $CD45.1^+$ cells (of donor origin) among resDCs and migDCs. Data were pooled from three independent experiments (n = 6-8). (**B**) Exemplary dotplots of subsets from resDCs and migDCs in endogenous mLNs and pLNs; data taken from two to three independent experiments (n = 12-14). (**C-E**) migDCs were isolated from indicated LNs. RNA-seq[L] and subsequent analysis was performed. (**C**) Colored numbers in scatterplots represent DEGs for the respective pair-wise comparisons of migDCs ($|\log_2(FC)| \geq 0.8$ and q-value ≤ 0.05). On the x-axis $\log_2(FC)$ of gene expression from migDCs (endogenous LNs) is plotted. On the y-axis $\log_2(FC)$ of gene expression from migDCs of transplanted mLN- versus pLN-SPF is plotted. (**D**) Heatmap of $\log_2(RPKM)$ values of marker genes; numbers in brackets indicate mean $\log_2(RPKM)$ over all replicates and samples. (**E**) GO analysis of biological processes for up- ('Maintained') or down-regulated ('Repressed') genes in migDCs isolated from transplanted mLN-SPF; numbers represent GO identifications. Data pooled from two to three independent experiments (n = 4-7). migDC, migratory dendritic cell ($Lin^-CD11c^+MHCII^{high}$); resDC, resident dendritic cell ($Lin^-CD11c^{high}MHCII^+$); Lin, $CD3^+CD45R^+Ly6G^+F4/80^{high}$.

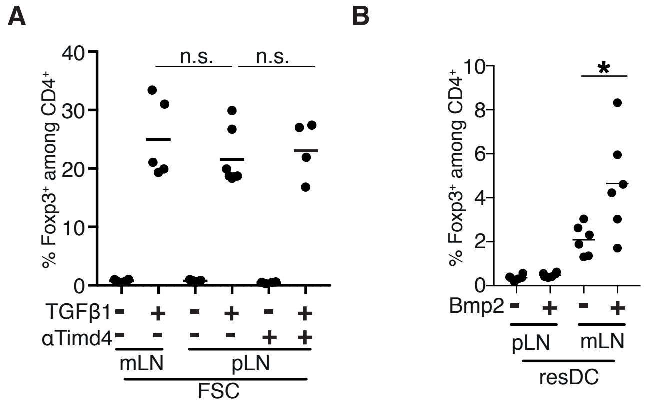

Supplementary Figure 7

Supplementary Figure 7: FSC and resDC Treg induction capacity. (**A**) Scatterplots show frequencies of Foxp3$^+$ Tregs among CD4$^+$ T cells after co-culture with FSCs obtained from indicated locations with or without supplementation of TGFβ1 (0.1 ng/ml) and/or αTimd4 (2.5 µg/ml) under polyclonal stimulation. (**B**) Scatterplots show frequencies of Foxp3$^+$ Tregs among CD4$^+$ T cells after co-culture with resDCs obtained from indicated locations with or without supplementation of exogenous Bmp2 (250 ng/ml). Data pooled from two to three independent experiments (n = 3-7). migDC, migratory dendritic cell (Lin$^-$CD11c$^+$MHCII^{high}); resDC, resident dendritic cell (Lin$^-$CD11c^{high}MHCII$^+$); Lin, CD3$^+$CD45R$^+$Ly6G$^+$F4/80^{high}
