# Supplementary material for: Neonatally imprinted stromal cell subsets induce tolerogenic dendritic cells in mesenteric lymph nodes
Source: Nat Commun. 2018 Sep 25;9:3903. doi: 10.1038/s41467-018-06423-7 (PMC6156403; doi:10.1038/s41467-018-06423-7)
Supplement: Supplementary file 3 — Description of Additional Supplementary Files [file 41467_2018_6423_MOESM3_ESM.pdf]

Supplementary Tables legends

Supplementary Table 1: DEGs between mLN- and pLN-derived FSCs independent of microbial colonization. $CD45^-Ter119^-CD31^-gp38^+$ FSCs were isolated from mLNs and pLNs of GF or SPF mice, and RNA-seq and subsequent analysis was performed. DEGs were identified in colonization (SPF vs. GF)- and location (mLNs vs. pLNs)-dependent pairwise comparisons ($|log2(FC)| \geq 1$ and q value ≤ 0.05). DEG, differentially expressed gene; GF, germ-free; FC, foldchange; mLN, mesenteric lymph node; pLN, peripheral skin-draining lymph node; RPKM, reads per kilobase of exon length per million mapped reads; SPF, specific pathogen-free.

Supplementary Table 2: mLN-specific transcriptional signature maintained in FSCs from transplanted mLN-SPF. $CD45^-CD24^-CD31^-gp38^+$ FSCs were isolated from endogenous mLN- and pLN-SPF, transplanted pLN- and mLN-SPF or transplanted mLN-GF. RNA-seq^L and subsequent analysis was performed. Table summarizes DEGs ($|log2(FC)| \geq 1$ and q-value ≤ 0.05) persistently up- or down-regulated in FSCs from transplanted mLN-SPF when compared to FSCs from transplanted pLN-SPF as well as endogenous mLN-SPF and pLN-SPF.

Supplementary Table 3: Transcriptional signature genes per stromal cell subset. Single cell suspensions from mLN-SPF and pLN-SPF were sorted for $CD24^-CD45^-$ cells and subjected to scRNA-seq. FSCs were identified as non-LECs, non-BECs as well as $Pecam1^-$ and $Ackr4^-$. Table summarizes marker DEGs up-regulated for each cluster. pct.1, percent of cells expressing gene in respective cluster; pct.2, percent of cells expressing gene in respective reference (1 = 100 %).

Supplementary Table 4: Transcriptional signature genes per mLN-SPF stromal cell subset. Single cell suspensions from mLN-SPF were sorted for $CD24^-CD45^-$ cells and subjected to scRNA-seq. FSCs were identified as non-LECs, non-BECs as well as $Pecam1^-$ and $Ackr4^-$. Table summarizes marker DEGs up-regulated for each cluster. pct.1, percent of cells expressing gene in respective cluster; pct.2, percent of cells expressing gene in respective reference (1 = 100 %).

Supplementary Table 5: Transcriptional signature genes per pLN-SPF stromal cell subset. Single cell suspensions from pLN-SPF were sorted for $CD24^{-}CD45^{-}$ cells and subjected to scRNA-seq. FSCs were identified as non-LECs, non-BECs as well as $Pecam1^{-}$ and $Ackr4^{-}$. Table summarizes marker DEGs up-regulated for each cluster. pct.1, percent of cells expressing gene in respective cluster; pct.2, percent of cells expressing gene in respective reference (1 = 100 %).
